# A Highly Standardized Pre-Clinical Porcine Wound Healing Model Powered by Semi-Automated Histological Analysis

**DOI:** 10.3390/biomedicines12081697

**Published:** 2024-07-31

**Authors:** Ives Bernardelli de Mattos, Alexandru C. Tuca, Fabian Kukla, Thomas Lemarchand, Danijel Markovic, Lars P. Kamolz, Martin Funk

**Affiliations:** 1 Department of Tissue Engineering & Regenerative Medicine (TERM), University Hospital Würzburg, 97080 Würzburg, Germany; ives.de_mattos@uni-wuerzburg.de; 2 EVOMEDIS GmbH, 8036 Graz, Austria; martin.funk@evomedis.com; 3 Department of Surgery, Division of Plastic, Aesthetic and Reconstructive Surgery, Medical University of Graz, 8036 Graz, Austria; lars.kamolz@medunigraz.at; 4 TPL Path Labs GmbH, A Stagebio Company, 79111 Freiburg, Germany; fkukla@stagebio.com (F.K.); tlemarchand@stagebio.com (T.L.); 5 Department of Biomedical Research, Medical University of Graz, 8036 Graz, Austria; danijel.markovic@medunigraz.at; 6 Joanneum Research Forschungsgesellschaft mbH, COREMED, 8036 Graz, Austria

**Keywords:** wound healing model, semi-automated analysis, standardization, reproducibility, burns, translational model

## Abstract

The wound-healing process is a physiological response that begins after a disruption to the integrity of tissues present in the skin. To understand the intricacies involved in this process, many groups have tried to develop different in vitro models; however, the lack of a systemic response has, until this day, been the major barrier to the establishment of these models as the main study platform. Therefore, in vivo models are still the most common system for studying healing responses following different treatments, especially porcine models, which share several morphological similarities to the human skin. In this work, we developed a porcine excisional wound model and used semi-automated software as a strategy to generate quantitative morphometric results of healing responses by specific tissues and compartments. Our aim was to extract the most information from the model while producing reliable, reproducible, and standardized results. In order to achieve this, we established a 7-day treatment using a bacterial cellulose dressing as our standard for all the analyzed wounds. The thickness of the residual dermis under the wound (DUtW) bed was shown to influence the healing outcome, especially for the regeneration of epidermal tissue, including the wound closure rate. The analysis of the DUtW throughout the entire dorsal region of the animals opened up the possibility of establishing a map that will facilitate the experimental design of future works, increasing their standardization and reproducibility and ultimately reducing the number of animals needed. Thus, the developed model, together with the automated morphometric analysis approach used, offers the possibility to generate robust quantitative results with a rapid turnaround time while allowing the study of multiple extra morphometric parameters, creating a more holistic analysis.

## 1. Introduction

The healing process of a cutaneous wound is dynamic and regulated by many mechanisms [1,2,3,4,5,6]. During this process, the tissues surrounding the wounded area play a pivotal role in the re-establishment of its integrity. For instance, cells in the remaining adjacent tissue offer support to the regenerating tissue and, together with the damaged extracellular matrix (ECM), contribute to giving signals to initiate immune and inflammatory cell infiltration [7] and guide the initial steps that will ultimately lead to the wound-closure process. Additionally, the treatment strategy influences the wound-healing outcomes. The decision to use an active moisture donor, for example, instead of a regular cotton gauze, requires a deep analysis of the tissue response [8]. Therefore, to better understand all the intricacies and study how to improve the healing outcomes, a suitable model is required.

In recent decades, research groups have combined efforts to follow the 3R guidelines for animal experiments first established in 1959 by Russell and Burch [9]. The 3Rs concept is based on replacing animals using alternative models, reducing the number of animals in tests, and refining procedures to avoid harmful methods that could cause distress to the animals. Various groups have already established effective and robust in vitro models to study skin wound healing [10,11,12,13,14]. As a tool to analyze the local response, these models have exceeded expectations; however, they lack the capacity to analyze a systemic response. Unfortunately, there is no available in vitro model capable of offering the possibility of studying the completeness of wound healing in all its intricate and complex events—notably, the influence of circulating cells such as immune or inflammatory cells on successful wound healing. Such a model would require the coordinated response of several tissues, including the systemic response, and culminating in a multifactorial process that ultimately promotes the closure of the opened wound [1,3,5,15]. Since the use of animal-based tests seems to be unavoidable, an effort aimed at the reduction and refinement of the procedures has become necessary. Some attempts to increase the data generated by in vivo wound models include multiplying the number of wounds [16]. This strategy creates a more painful experience for the animals, which must be considered during the elaboration of the analgesic method. To refine the procedure, a more standardized and reproducible approach could be a better alternative.

Thus, this study proposes the development of an in vivo excision wound model with improved standardization and reproducibility while following a strict program to minimize the distress caused to animals. To this end, we performed a deep analysis of the residual dermis for each dorsal site position and its influence on several healing parameters. Based on the observed dermal morphology, we have created a wounding map to aid future work and provide guidance to improve standardization and reduce the number of animals required. Additionally, the described model is powered by semi-automated histological analysis software that allows for agile and reliable morphometric data generation while opening up the possibility of including far more morphological parameters that are often neglected.

## 2. Material and Methods

### 2.1. Animal Procedure

The animal procedures were approved by the Animal Care and Use Committee (Veterinary University Vienna, Austrian Ministry of Science and Research), as stated in document number BMBWF-66.010/0018-V/3b/2019. All animals were treated in accordance with the recommendations of GV SOLAS (Gesellschaft für Versuchstierkunde/Society of Laboratory Animal Science, Germany). A total of eight female domestic pigs (*Sus domesticus*; hybrid from *Deutsche Landrasse* and *Deutsches Edelschwein*) aged 3–4 months old and weighing between 34 and 47 kg at the start of the experiments were housed in 12:12 light–dark cycles in neighboring and individual indoor stalls with controlled temperature (22 °C ± 2 °C) and humidity (55–65%). The animals received daily rations of the G-22 swine feed formulation (Gsellmann Mischfuttererzeugung GmbH, Kohlberg, Austria) and had water access ad libitum. Food access was withdrawn 12 h prior to the procedure. Pre-emptive analgesia using 0.5 mg/kg midazolam (Dormicum^®^, Roche Austria GmbH, Vienna, Austria), 2 mg/kg azaperon (Stresnil^®^, Elanco, Sanochemia Pharmazeutika AG, Neufeld an der Leitha, Austria), and 10 mg/kg ketamin (Ketasol^®^, aniMedica GmbH, Frankfurt, Germany) was given to the animals together with pre-oxygenation for 5 min using 100% oxygen. Upon arrival in the operating room, 2–5 mg/kg of propofol bolus (Propofol, Fresenius Kabi Austria GmbH, Graz, Austria) was administrated through a venipuncture to the marginal ear vein of the animal.

**Figure 1:**
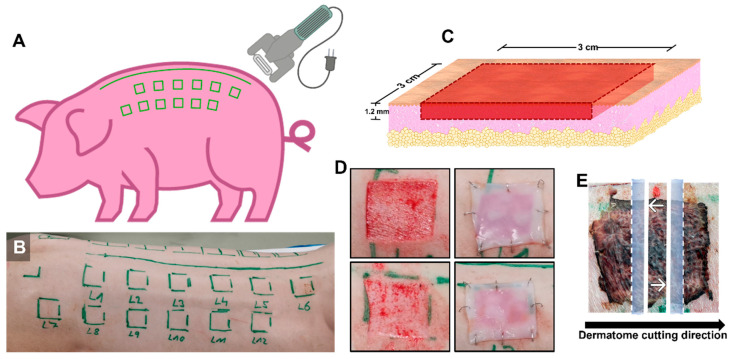
Establishment of the wound donor sites and guide for the preparation of the histological slides. (**A**) Schematic illustration depicting the region on the dorsum of the animals where the wounds would be generated. (**B**) Image of the dorsum of a representative example of how the areas would be delineated. (**C**) Illustration depicting how the excision wounds would be created. (**D**) Images showing two examples of freshly created wounds and wounds after the placement of the wound dressing. (**E**) Visual guide for histological section preparation White arrows indicating the position where the section sequence started.

The wounds were established equidistantly 3 cm from the spine, with one dermatome used to obtain skin grafts (Figure 1). A wounding system encompassing 24 wounds, 12 on the left flank of the dorsum and 12 on the right flank, was used. The animals were positioned in ventral recumbency. The dorsal area had previously been shaved using an electric razor, and the skin surface was cleaned using a cloth soaked in Octenisept^®^ (Schülke and Mayr GmbH, Vienna, Germany) solution. Twenty-four areas of 3 × 3 cm were delineated on the dorsum of the animals prior to the excisions (Figure 1A,B). Wound number 1 was created adjacent to the animal scapula and marked right after the bone position. The subsequent wounds were generated with 3 cm spaces in between. For anatomical reasons, wound number 12 was created 3 cm under wound number 5, creating 2 staggered rows. All the following wounds (7–11) were then generated following the same strategy as explained previously, with wound number 7 positioned over the animal scapula. The dermatome wounds were set to a 3 × 3 cm size and a 1.2 mm depth and were generated in the marked regions (Figure 1C). During the procedure, the animals received oxygen through a ventilation mask and, depending on the necessity, 1–2% sevoflurane (SEVOrane^®^, AbbVie GmbH, Vienna, Austria) was administrated using the same inhalation method. Additionally, to avoid the desiccation of the conjunctiva, Oleovit^®^ (Fresenius Kabi AG, Bad Homburg, Germany) was used. Several vital signs were monitored: pulse oximetry, electrocardiogram, capnography, and body temperature. Continuous analgesia was achieved through the application of an opioid transdermal patch (fentanyl 50 µg/h; Gebro Pharma GmbH, Fieberbrunn, Austria). This patch was changed every 48 h post-procedure. The animals also received an infusion of isotonic solution (Elomel 5–10 mL/kg/h; Fresenius Kabi AG, Bad Homburg, Germany). The animals were assessed daily for signs of pain or any type of distress by the attending veterinarians.

As a primary wound dressing, a commercially available bacterial nanocellulose (BNC; epicite^hydro^, QRSkin GmbH, Wuerzburg, Germany, Ref-No. 800003-M02B) dressing was used. The BNC used in the treatment had previously shown consistent results [8,17,18] and offered an interesting moisture balance to support the healing process. The BNC dressing was cut into 5 × 5 cm pieces and placed on the wounded area to cover it completely. The dressing was secured in place using surgical staples (Weck Visitat^®^, Teleflex Medical, Morrisville, NC, USA) (Figure 1D). Fatty gauze (JELONET^®^; Smith and Nephew GmbH, Munich, Germany) was used as the secondary dressing, with cotton gauze placed on top of the dressing to cover and protect the BNC and the secondary dressing. After the procedure, the animals received a medical recovery suit (Buster body suit for dogs, size large, Jorgen Kruuse A/S, Langeskov, Denmark) designed for pets to protect the animals and to maintain the dressings in place.

After 7 days, the animals were euthanized under deep anesthesia and analgesia using a 1 mol/L dose of potassium chloride (1 M-Kaliumchlorid-Lösung, Fresenius Kabi AG, Germany). Photo documentation of the tissue/wound dressing sampling was performed for further analysis. A 5 × 5 cm area of tissue was excised from each wound above the superficial fascia layer and placed in super-mega cassettes (Diapath S.p.A., Bergamo, Italy). All the wound specimens were then stored in histological containers filled with neutral buffered formalin and sent to TPL path Labs GmbH (Freiburg, Germany), where the tissue blocks were trimmed transversely, including the subcutis/panniculus, dehydrated, embedded in paraffin, and microtome-cut at a nominal thickness of 3 μm. The obtained sections were mounted on standard glass slides and stained with hematoxylin and eosin (H&E) according to standard operating procedures. A guide to specify where the sections should be taken was provided. The sections were generated at the central part of the wounded area and cut perpendicular to the dermatome cutting direction to ensure less width variation (Figure 1E).

In total, two slides were created in two different regions of the wounded area. The rationale for choosing two section levels was based on the observation of wound healing heterogeneity. The slides were prepared, including the entire wounded area and the adjacent unwounded tissue on the left and right parts of the wound edge. Unwounded tissue under the wound bed was also included as part of the overall thickness transverse trimming. All H&E-stained sections were digitized using an Axioscan Z1 (Zeiss Microscopy, Jena, Germany). The estimated analyzed area ranged roughly from 5.0 × 10^7^ to 7.0 × 10^7^ µm^2^ per histological cut. The wounded area was then analyzed by a histopathologist. For each created wound, the repair process of two main tissue types was analyzed: the epidermal tissue (regenerated new epidermis area, percentage of re-epithelialization, and average thickness) and the dermal tissue (new dermal tissue: granulation tissue area and average thickness). Using automated VISIOPHARM^®^ software version 2020.08 (Visiopharm A/S, Hoersholm, Denmark), the aforementioned healing parameters were analyzed, and morphometric data were generated. Additionally, to maximize the information extracted from the histological slides, an analysis of extra parameters, such as the exudate area present on the surface of the wound at day 7 and the standard thickness of the primary wound dressing, was also performed. Manual measurements of the morphological characteristics of the remaining dermis were also carried out using Zen 3.3 blue edition software (Zeiss Microscopy, Jena, Germany) to define the “ground truth” and validate the automated analysis.

### 2.2. Statistical Analysis

The results are expressed as the mean ± standard deviation (SD). Statistically significant differences were calculated using Student’s *t*-test and ordinary one-way ANOVA, with significance indicated by a *p*-value less than 0.05. Pearson’s correlation analysis was calculated to analyze the relationship between the different healing parameters and the morphometric characteristics of the tissue. The results are expressed in Pearson’s correlation coefficients (r), 95% confidence intervals, and *p*-values. Simple linear regression was calculated and plotted as a guide to help observe the tendency of the scattered plots. Data analysis was calculated using GraphPad Prism 9 (GraphPad Software, San Diego, CA, USA).

## 3. Results

### 3.1. Morphometry Measured Using Semi-Automated Software

**Figure 2:**
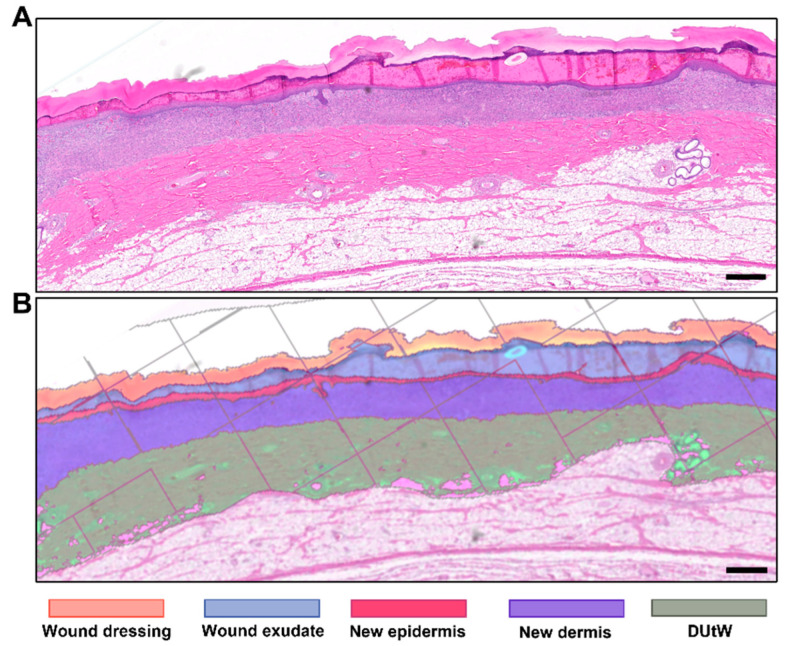
Tissue recognition using automated software. Representative example of the morphometric analysis at day 7 of the areas of the wound dressing, the exudate on the wound surface, the new epidermis, the new dermis, and the dermis under the wound (DUtW), performed by the automated software. (**A**) Microscopy of an H&E-stained slide. (**B**) Image of the same microscopy postanalysis by the software. The scale bar represents 500 μm.

After the preparation of the H&E-stained slides, scanned digital imaged sections (whole-slide imaging) were analyzed using automated system software. The different components present in the slides were recognized by the software and labeled accordingly (Figure 2). Using an artificial intelligence approach—specifically, a deep neural network algorithm—the software was capable of recognizing five different patterns: the original dermal tissue remaining under the wound bed; the new regenerated dermal tissue, owing to a markedly different texture cell content and tinctorial affinities; the newly regenerated epidermal tissue; the wound exudate; the wound dressing covering the wound; and the empty spaces/subcutis. In addition, the software recognized normal or dilated epithelial skin adnexa (sweat glands, sebaceous glands, and a few hair follicles), which were excluded from the “new dermal tissue” class. After the delimitation of the components, the software provided different measurements, including the area of the labeled segment. The wound width (i.e., the wound’s contoured length from one wound edge to the other edge) was framed manually due to complex criteria to determine the wound edges. Usually, the limit is set at the beginning of a visible new dermal tissue and/or epidermis within normal limits beyond the hyperplastic ridges at the edge of the wound. Using this value, the average thickness of the regenerated new dermal tissue (area of new dermal area over wound width), as well as the percentage of re-epithelialization (length covered by the new epithelium over the wound width), was obtained.

To validate the method, the wound width (dermal + epidermal widths obtained separately) was measured manually on a subset of 20 wound site sections using two methods: the standardized dermal and epidermal thicknesses (the respective manually contoured areas divided by wound length) and the estimated dermal and epidermal wound thickness with 20 randomly chosen linear vertical measurements to determine the ground truth.

### 3.2. Remaining Dermis under the Wounded Area

**Figure 3:**
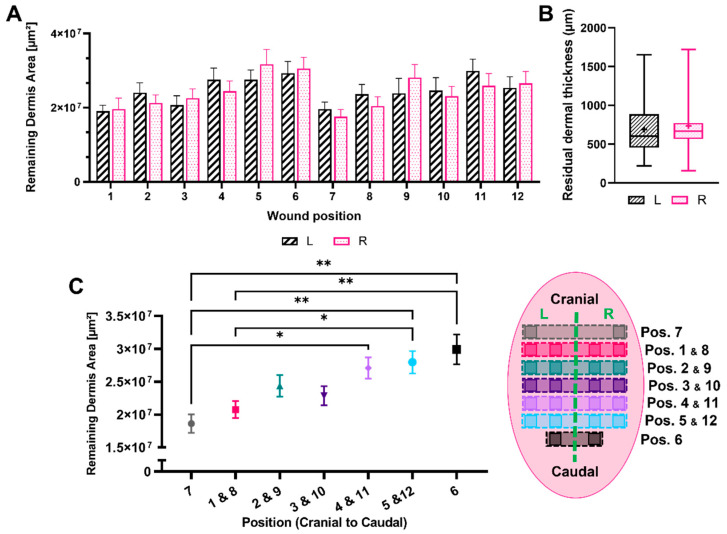
Area of the remaining dermis under the wound. Diagram showing (**A**) the results of the remaining dermis under the wound (DUtW) area for each position, left and right, and (**B**) the results comparing the thicknesses of the DUtW for positions left and right combined. (**C**) Diagram for the areas of the remaining DUtW for each position and schematic representation of the donor site position scattered on the dorsal part of the animals (*n* = 8). The results are expressed as means and standard errors of the mean. *p* * < 0.02 and *p* ** < 0.009.

Using the quantitative morphometric data generated by the semi-automated software, an extensive analysis of the remaining DUtW was conducted (Figure 3). A total of 321 measurements were generated from eight animals, with an average of two slides analyzed per wound. A cranial–caudal position-dependent variation for this morphometric parameter was noticed, with no substantial difference observed when comparing matching left–right opposite positions in both flanks of the animal (Figure 3A). In addition, no significant differences were observed when comparing all the wounds generated on the left flank with those generated on the right flank, thus showing consistent variation (Figure 3B). When the wounds were grouped based on their dorsal positions, the wounds generated closer to the caudal part of the dorsum presented higher values for the remaining DUtW (Figure 3C).

**Figure 4:**
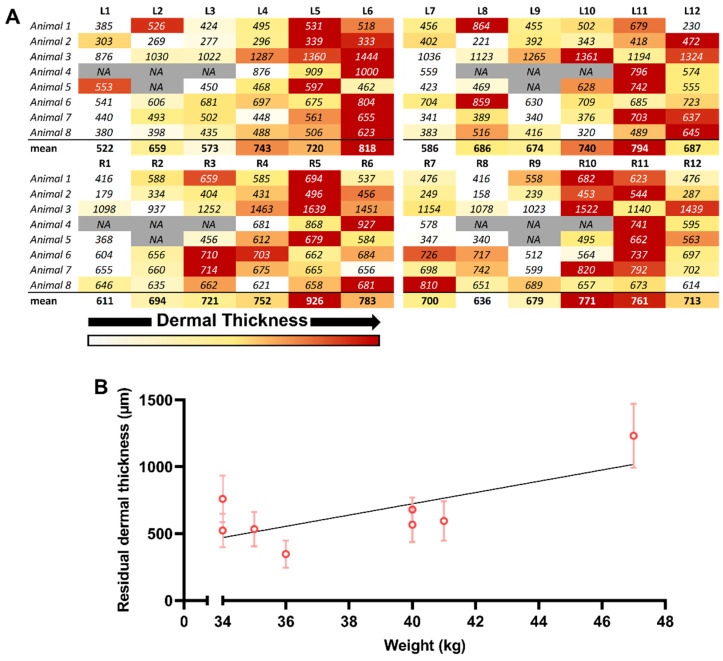
Individual animal influence on the thickness of the remaining dermis under the wound (DUtW). (**A**) Heat map of the DUtW average thickness (μm) for each studied animal, considering the dorsal flank where the donor sites were created. The DUtW values were arranged considering positions 1–6 and positions 7–12. The burgundy tone represents high values, the ocher tone represents mid-to-high values, the yellow tone represents mid values, the light yellow tone represents mid-to-low values, and the white tone represents low values. In grey boxes, NA represent sections where the measurements for this area was not possible. (**B**) Correlation between the residual dermis thickness and the body weight of each animal. The results are expressed as means and standard deviations. Simple linear regression was calculated and plotted as a guide to help observe the tendency of the scattered plots, with no statistical importance.

Following this analysis, we decided to study this morphological variation in DUtW areas in a more individualized way (Figure 4). Therefore, a heat map was built for each individual site of each animal using the mean value of the thickness of the DUtW for each individual site (Figure 4A). In addition, the mean values of the dermal thickness for each position were calculated, and a heat map was produced. Here, it was possible to observe that the previously reported wide thickness variations were more consistent considering each animal alone, which could suggest the influence of body size on this parameter. The relationship, if any, between the thickness of the DUtW and animal body weight was consequently studied, revealing a strong and positive correlation (r = 0.72, confidence interval, 0.04–0.95, *p* = 0.04) [19] (Figure 4B).

**Figure 5:**
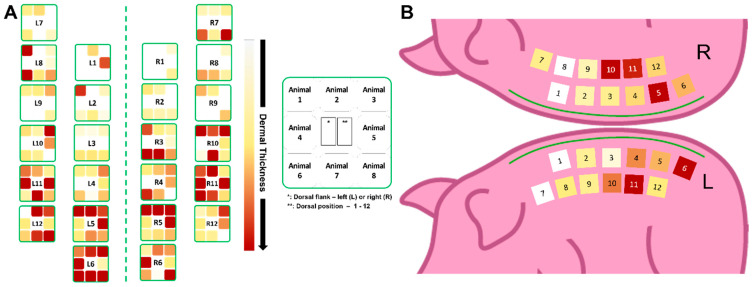
Schematic map of the thickness of the dermis under the wound. (**A**) Schematic illustration of the individual mean values for the thickness of the dermis under the wound (DUtW) obtained for each position. Highlighted is the model depicting where the results for each of the eight animals were plotted. (**B**) Final map compiling the mean results for the thickness of the DUtW.

The heat map pattern obtained was then used to produce a guide indicating the expected thickness for each dorsal region (Figure 5). First, the individual results obtained for each position were plotted in a schematic map (Figure 5A). As observed in the previous results, positions close to the caudal part of the animals presented higher mean values for the thickness of the DUtW, with darker tones being more present in this region. Lighter tones were more recurrent in the region close to the cranial part. A final map enclosing the mean values for each position of all the animals combined was established (Figure 5B).

**Figure 6:**
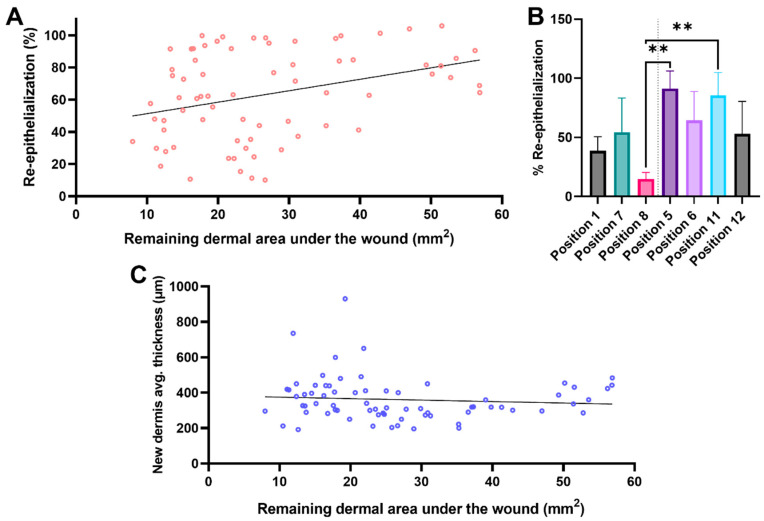
Influence of the remaining dermis under the wound on different healing parameters. (**A**) Scatter plot with morphometric data for the correlation between the percentage of re-epithelialization and the area of the dermis under the wound (DUtW). (**B**) Bar diagram of the percentage of re-epithelialization comparing the results for positions closer to the cranial part of the dorsum (positions 1, 7, and 8) and closer to the caudal part (positions 6, 11, and 12). *p* ** < 0.008. (**C**) Scatter plot of the correlation between the average thickness of the new dermal tissue and the area of the remaining DUtW. Simple linear regression was calculated and plotted as a guide to help observe the tendency of the scattered plots, with no statistical importance.

Subsequently, the analysis of several healing parameters was studied in light of the morphometric results obtained for the DUtW (Figure 6). Using the BNC as the primary dressing due to its healing support capacity, assessments for the regenerative process in the epidermal and the dermal tissue were analyzed. Considering the re-epithelialization of the wounded area (Figure 6A), a moderate and positive correlation was obtained (r = 0.34, confidence interval: 0.12–0.53, *p* = 0.004), indicating that the cells present in the remaining DUtW offered direct (if no or very few new dermises) or indirect (if new dermises) support to the keratinocytes, promoting more successful wound closure. Looking deeper into this influence, we decided to compare the re-epithelialization results in wounds generated near the cranial part of the dorsum to those close to the caudal part (Figure 6B). Interestingly, although the difference was not always statistically significant, it was possible to observe a positive tendency in which the areas closer to the caudal part, which showed a thicker DUtW, performed slightly better than their counterparts closer to the cranial part. When the measurements for the regenerated dermal tissue were analyzed, no significant correlation was observed, although the scattered data could indicate a tendency toward a negligible correlation (r = −0.09, confidence interval: −0.32–0.15, *p* = 0.46) (Figure 6C).

**Figure 7:**
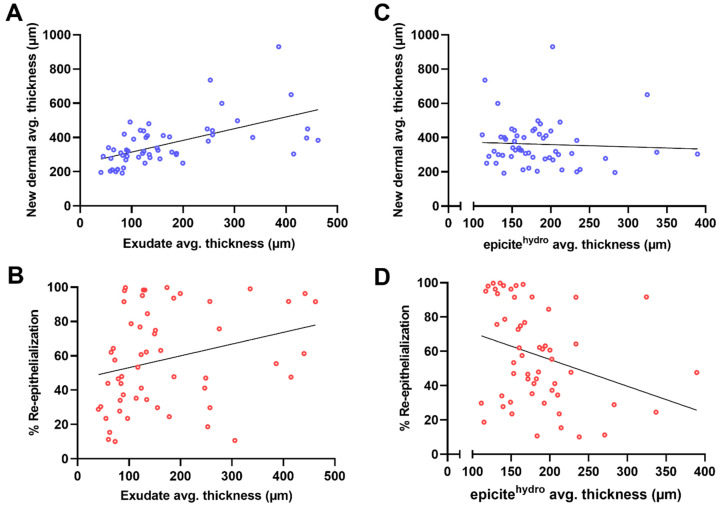
Influence of exudate and moisture balance on regenerated dermal tissue and wound closure. (**A**) Scatter plot of the morphometric data for the average thickness of the exudate after 7 days of treatment correlated to the average thickness of the new dermal tissue. (**B**) Diagram with the scatter plot of the correlation between the average thickness of the exudate and the percentage of re-epithelialization. (**C**) Scatter plot of the average thickness of the epicite^hydro^ dressing after the 7-day treatment correlated with the average thickness of the regenerated dermal tissue and (**D**) correlated with the re-epithelialization rate. Simple linear regression was calculated and plotted as a guide to help observe the tendency of the scattered plots, with no statistical importance.

Other important factors could also be explored using this model (Figure 7). For instance, the influence of the presence and amount of exudate present on the surface of the regenerated tissue could be studied through morphometric analysis (Figure 7A,B). The average thickness of the exudate, when correlated to the regenerated dermal tissue, showed a positive and moderate relationship (r = 0.57, confidence interval: 0.37–0.73, *p* < 0.0001). When the same parameter was correlated with the wound-closure process, a weak and positive relationship was obtained (r = 0.28, confidence interval: −0.01–0.50, *p* = 0.04). These results indicate that the dermal tissue regeneration seemed to be more affected by the volume of exudate at the end of the 7-day treatment period. By measuring the thickness of the BNC dressing at the end of the same period, an inference related to the moisture balance in the wound milieu could be drawn (Figure 7C,D). The thicker the dressing, the longer the period when the wound had a highly hydrated environment and vice versa. Considering the correlation between the average thickness of the BNC dressing and the average thickness of the regenerated dermis, no significant relationship was observed (r = −0.05, confidence interval: −0.32–0.21, *p* = 0.69), although a tendency toward a negligible correlation was observed in the scattered plot. When the correlation was calculated between the dressing thickness and the re-epithelialization rate across the wound surface, a moderate and negative relationship was obtained (r = −0.30, confidence interval: −0.53 to −0.04, *p* = 0.02).

**Figure 8:**
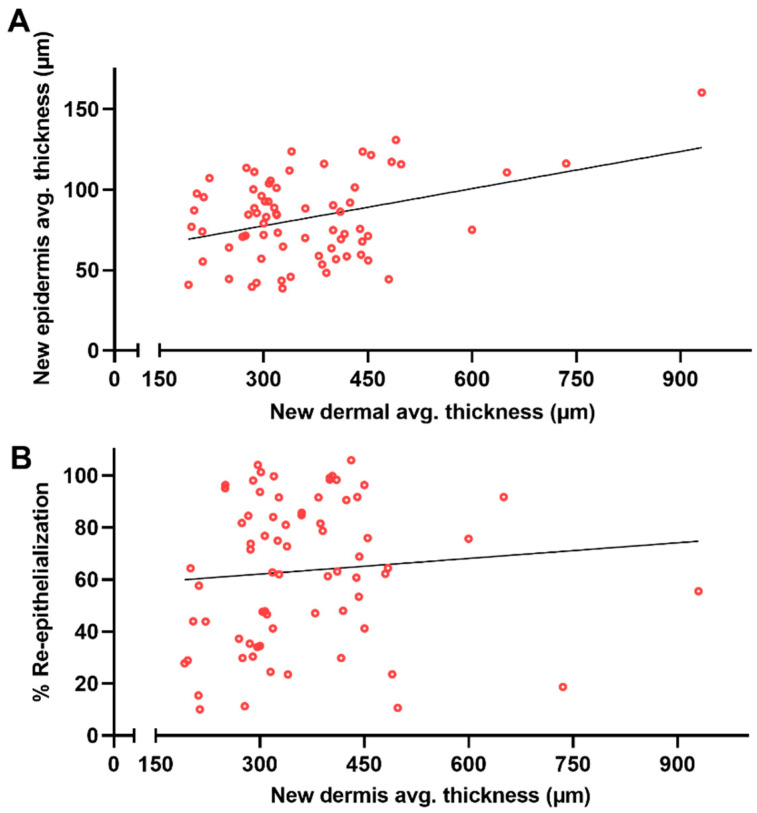
Influence of regenerated dermal tissue on different epidermal healing parameters. (**A**) Scatter plot of the average new dermal thickness correlated with the new epidermal area and (**B**) correlated with the percentage of re-epithelialization. Simple linear regression was calculated and plotted as a guide to help observe the tendency of the scattered plots, with no statistical importance.

Since the remaining dermal tissue present under the wound bed appeared to not offer support for the regeneration of the new epidermal tissue, we decided to test whether the newly regenerated dermal tissue influenced different epidermal regenerative parameters (Figure 8). Therefore, the relationship, if any, between the average thickness of the regenerated dermis and both the average thickness of the new epidermis and the wound closure rate (percent of new epithelium) was studied (Figure 8A and Figure 8B, respectively). Correlation tests indicated a positive and moderate relationship with the average thickness of the regenerated epidermis (r = 0.37, confidence interval: 0.15–0.55, *p* = 0.002), while no correlation with the percentage of re-epithelialization was observed (r = 0.09, confidence interval: −0.15–0.32, *p* = 0.46). Table 1 summarizes the obtained results.

## 4. Discussion

In an attempt to offer a transparent report, we followed the recommended guidelines presented in the last version of the ARRIVE (2.0), an important guideline for reporting results using animals [20]. The utilization of a morphometry analysis based on automated software significantly reduced the time between the slide analysis and data acquisition. Previously, morphometric analysis was performed through a laborious process in which each tissue was delineated by hand using computer software. The time needed to generate a complete set of data was considerable, taking many weeks until an entire analysis was performed. The automated analysis, on the other hand, offered an expeditious process, with entire datasets generated in a 24 h time frame, once the algorithm applications had been validated for sensitivity and accuracy. Additionally, due to the nature of the process, high standardization can be achieved using this approach, since intrinsic human error can be avoided. It is important to highlight that the generated results were extremely similar to those obtained using the previous method (data to be published), which is crucial to maintain the same quality level of the analysis. Hence, the tissue recognition promoted by the automated software offered a fast, high-resolution analysis while enhancing the standardization and reproducibility of the model, which per se also increased its reliability. Using this method of analysis, the opportunity to explore other parameters was opened. The morphometric analysis of the exudate present on the tissue surface at the end of the treatment and the average thickness of the wound dressing are two examples. In the future, and with the further development of the technology, other wound-healing parameters could be analyzed, especially in combination with other histological staining techniques.

A multitude of wound models using pigs have already been described in the literature (Table 2) [21,22,23,24,25]. Burn and excisional wound models have been extensively used to study partial, deep-partial, and full-thickness wounds [21,22,23,24,26,27,28,29,30,31,32,33,34,35,36,37]. However, most works have relied solely on the analysis of the wound closure process [21,38], and the vast majority have focused only on the regenerated tissue, excluding the possible influence caused by the surrounding tissue on the healing outcome. Here, we tried to include as many morphometric parameters as possible to achieve a holistic analysis. Additionally, the decision for an excisional approach was to increase the reproducibility and avoid uncontrollable effects that could lead to lesser standardization (e.g., burn wound conversion [39]). Despite the decision for an excisional model, the model described here can still be used to study burns, since deeper burns often require surgical intervention to eliminate damaged tissue [40,41]. Moreover, excision models are regularly used to test the medical devices used in the treatment of burns [22,23,24].

Our model used 24 wounds generated on the dorsum of the animals. Using Kelley’s equation to calculate the total body surface area [44], the excised area varied between ~2.34% and ~2.54% of the total body surface, representing only a small and ethically acceptable fraction of the skin surface of the animal. Through an analysis of the average thickness of the DUtW, it was possible to observe variations depending on the animal’s weight and the position of the donor site. As expected, the dermal tissue was thicker in the larger animals. Interestingly, the dermal tissue on the dorsum of the animals varied significantly depending on the cranial–caudal position. For instance, the dorsal dermis closer to the cranial part of the animal was thinner, while the dermal area increased as the wounded site was positioned closer to the caudal part. These results are particularly interesting due to the level of influence that the remaining dermis had on the healing parameters. We were able to show that the DUtW positively impacted, to a certain level, the re-epithelialization extent of the wounded area. Using the same treatment, the wound closure rates for wound sites closer to the cranial part of the dorsum were statistically poorer in comparison to those closer to the caudal part. This tendency was not only observed for the same animals, but was also statistically significant for all animals used in these experiments. Therefore, future work relying on the analysis of the wound closure rate should also use a methodological approach, considering where the tested groups should be placed on the animal dorsum. These results have important implications for the study design in terms of using the most homogenous cohort according to age/weight and randomization with respect to the site map assignment. Using the data generated here, we were able to elaborate a dorsal map on which wounds with comparable DUtW were marked. This will facilitate the experimental design of works that decide to use a similar approach.

Conversely, the same effect was not observed for the regenerated dermal tissue. The regeneration of the dermis was not directly correlated with the remaining DUtW. Recently, our group showed that dermal tissue regeneration is heavily impacted by the moisturization of the wound bed [8]. Thus, when dermal regeneration was analyzed in correlation with the presence of exudate at the end of the treatment, a positive level of influence was observed. Considering that the exudate impacts, among other factors, the moisture balance of the wound milieu, this result is congruent with the previously reported results. The same influence was not observed for the percentage of re-epithelialization, which was also in agreement with the published results mentioned [8]. When the thickness of the wound dressing was analyzed, the moisture present on the last day of the treatment showed a low impact on the new dermal tissue and negatively influenced the wound closure rate. Once again, these results are in agreement with the aforementioned work published previously by our group. The wounds in this study were treated with a nonocclusive secondary dressing, which showed a relatively lower influence on dermal regeneration. Furthermore, wound closure for wounds showing a thicker epicite^hydro^, which corresponds to a highly moisturized environment, performed more poorly than wounds showing lower values for the thickness of the BNC dressing at day 7 [8].

In light of the presented results, we decided to explore the effect of the newly regenerated dermis on the epithelia. It was possible to observe that the thickness of the regenerated epidermis was impacted by the average thickness of the new dermal tissue, while no influence was obtained for the wound closure rate. To our knowledge, no other published work has performed such a comparative analysis.

## 5. Limitations

Although the tests were performed using animals within a fixed weight and age range, the map and the data generated could be extrapolated to heavier animals from the same species. As of this moment, the proposed model is applicable solely to the pig species described. Future works using the same method will be able to add more information regarding the morphological characteristics of other species, increasing knowledge and leading to standardization and reproducibility for other swine models.

## 6. Conclusions

Using automated software capable of analyzing several morphometric parameters, our group was able to generate reliable, standardized, and reproducible results in a 24 h time window. This expeditious analysis opens up the possibility of exploring some healing parameters that are often neglected. This method has, therefore, the potential to ultimately increase the amount of data extracted by each in vivo experiment while reducing the number of animals required. Furthermore, we were able to explore the influence of morphological aspects on the healing process. Since the wound closure rate is one of the major morphometric factors explored by groups studying new wound dressing strategies, we showed that experimental designs must consider the effect of the remaining DUtW on this parameter to improve the quality of the analysis. To our knowledge, this is the first work to address such characteristics. In the reported results, our group was able to show that the re-epithelialization rates for wounds treated with the same dressing performed significantly differently depending on the dorsal position where the wounds were generated. Dermal regeneration, on the other hand, was not directly impacted in the same way. However, for the dermis, we showed that the moisture of the wound bed played a more significant role. Additionally, we observed a certain level of influence that the regenerated dermis had on the regeneration of the epidermis. All the aforementioned results allowed for the creation of a wounding map with the potential to be used as a guide to help future work to elaborate a more standardized experimental design, positioning different treatments and controls in wounded sites that possess similar DUtW areas.

## Figures and Tables

**Table 1:** Compilation of the calculated Pearson’s correlation results between the analyzed morphometric parameters. The results are presented as Pearson’s coefficient r at a 95% confidence interval.

	Re-Epithelialization (%)	Thickness of the Regenerated Dermal Tissue (µm)
remaining dermal tissue (mm^2^)	0.34 * 0.12 to 0.53	−0.09 −0.32 to 0.15
thickness of the regenerated dermal tissue (µm)	0.09 −0.15 to 0.32	-
thickness of the exudate at the wound surface (µm)	0.28 * −0.01 to 0.50	0.57 * 0.37 to 0.73
thickness of the wound dressing (residual moisture) (µm)	−0.30 * −0.53 to −0.04	−0.05 −0.32 to 0.21

* Statistically significant (*p* < 0.05) for Pearson’s analysis.

**Table 2:** Comparison of excisional porcine wound models.

	No. of Animals	No. of Wounds per Animal	Pig	Weight Range (kg)	Wounded Area	Wound Depth	Healing Parameters	Histological Evaluation
Singer et al. (2003) [21]	3	Avg.: 38.3 (total 115)	*Sus scrofa domestica*	20–30	2.5 × 2.5 cm (6.25 cm^2^)	0.6 mm (SPT)	Wound closure and thickness of scar	Manual morphometric analysis
Singer et al. (2007) [42]	2	10	*Sus scrofa domestica*	40	2.5 × 2.5 cm (6.25 cm^2^) Ventral area	0.6 mm (SPT)	Wound closure	Manual morphometric analysis and optical coherence tomography
Wlaschin et al. (2019) [34]	9	4	*Sus scrofa domestica* Yorkshire Chester White crossed swine	28–32	2.5 × 2.5 cm (6.25 cm^2^)	0.5 mm (SPT)	Wound closure, serocellular crust, and epidermal thickness	Manual morphometric analysis
Schiefer et al. (2019) [24]	6	3	Minipigs	25.8 (±2.5)	2.4 × 2.4 cm (5.76 cm^2^)	0.5 mm (SPT)	Epidermal thickness and cutometer analysis	Manual morphometric analysis
Pirone et al. (1992) [25]	6	12	Five-way Cross swine	15–20	2.2 × 2.2 cm (4.84 cm^2^)	0.5 mm (SPT)	Epidermal wound healing and moisture vapor transmission rates	None
Travis et al. (2014) [43]	2	3	Male *Sus scrofa domestica* Duroc swine	30–55	7.6 × 7.6 cm (57.76 cm^2^)	~1.5 mm (DPT)	Wound closure and perfusion units	Manual morphometric analysis and laser doppler imaging
Connolly et al. (2020) [42]	16	6	*Sus scrofa domestica*	10–15	5 × 5 cm (25.00 cm^2^) Ventral area	0.1 mm (SPT)	Wound closure, epidermal hyperplasia, epidermal/dermal separation, inflammatory cells, hair follicles, glands, elastic fibers, smooth muscles, collagen orientation, fibroplasia, vascular proliferation, and hemorrhage	High-frequency ultrasound and manual subjective analysis
Kuo et al. (2022) [33]	Not described	20/19	*Lanyu pigs (Minipig)*	25	2.5 × 2.5 cm (6.25 cm^2^)	2.3 (DPT) and 6 mm (FT)	Wound closure, region-based variation on epidermis and dermis thickness, region-based variation on wound closure and contraction, tissue maturation after 2 and 6 months, blood flow, and collagen content	Manual morphometry, imaging software analysis, and laser doppler imaging
Current approach	8	24	*Sus domesticus:* hybrid from *Deutsche Landrasse* and *Deutsches Edelschwein*	34–47	3 × 3 cm (9.00 cm^2^)	1.2 mm (DPT)	Wound closure, epidermal area, epidermal thickness, regenerated dermis area and thickness, residual dermal area and thickness, area and thickness of exudate at day 7, and area and thickness of wound dressing at day 7	Semi-automated analysis

SPT: superficial-partial thickness, DPT: deep-partial thickness.

## Data Availability

The data presented in this study that support the findings will be made available on request by the corresponding author.
